# 688. Acute respiratory illness in pediatric hematopoietic stem cell transplant (HSCT) recipient's vs immunocompetent children: A multicenter analysis

**DOI:** 10.1093/ofid/ofaf695.227

**Published:** 2026-01-11

**Authors:** Ruby Sangha, Samar Musa, Raymond Pomponio, Jonathan Albert, Janet A Englund, Jennifer E Schuster, Geoffrey A Weinberg, Christopher J Harrison, Leila C Sahni, Eileen J Klein, Flor M Munoz, Julie A Boom, Rangaraj Selvarangan, Natasha B Halasa, Laura S Stewart, Peter G Szilagyi, Mary A Staat, Leah Goldstein, Heidi L Moline, John Williams, Marian G Michaels

**Affiliations:** UPMC Childrens Hospital of Pittsburgh, Pittsburgh, PA; University of Pittsburgh, Pittsburgh, Pennsylvania; University of Pittsburgh, Pittsburgh, Pennsylvania; UPMC Childrens Hospital of Pittsburgh, Pittsburgh, PA; Seattle Children’s Hospital/Univ. Washington, Seattle, Washington; Children's Mercy Kansas City, Kansas City, MO; University of Rochester Sch Med & Dent, Rochester, New York; Children's Mercy Hospital, Kansas City, Missouri; Baylor College of Medicine and Texas Children's Hospital, Houston, Texas; Seattle Children's Hospital and University of Washington School of Medicine, Seatte, Washington; Baylor College of Medicine Houston, Dallas, Texas; Baylor College of Medicine, Houston, Texas; Children’s Mercy Hospital, Kansas City, Missouri; Vanderbilt University Medical Center, Nashville, TN; Vanderbilt University School of Medicine, Nashville, Tennessee; UCLA, Los Angeles, California; Cincinnati Children's Hospital Medical Center, Park Hills, Kentucky; Centers for Disease Control and Prevention, Atlanta, Georgia; US-CDC, Atlanta, Georgia; University of Wisconsin, Madison, Wisconsin; University of Pittsburgh/ CHP, Pittsburgh, Pennsylvania

## Abstract

**Background:**

Children undergoing hematopoietic stem cell transplantation (HSCT) are at elevated risk for severe acute respiratory infections (ARI). We compared clinical outcomes between HSCT recipients and immunocompetent children with medically attended ARI using CDC’s New Vaccine Surveillance Network data.

**Figure:**
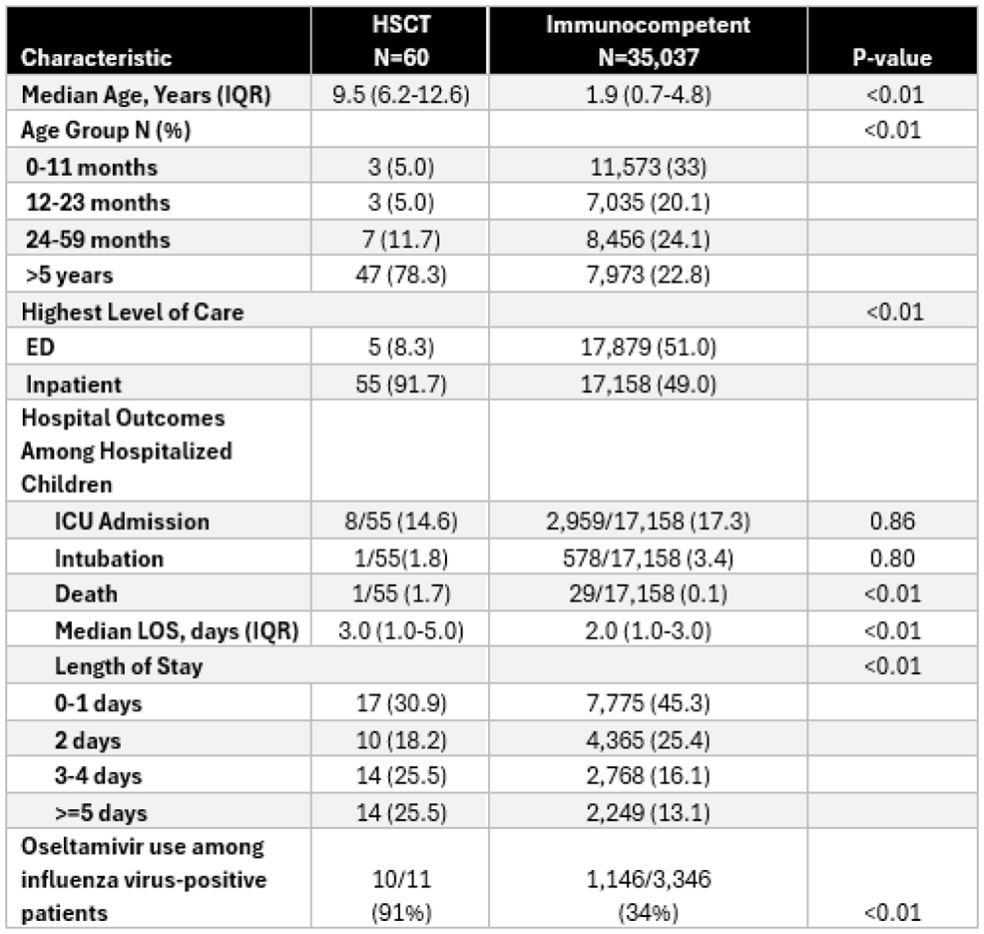
Comparison of Hematopoietic Stem Cell Transplant (HSCT) Recipients vs. Immunocompetent Children with Medically Attended Acute Respiratory Illness, New Vaccine Surveillance Network (NVSN), Dec 2016 – Sep 2020 Abbreviations: IQR = Interquartile range, LOS = Length of Stay Comparisons were made using Chi-squared tests for categorical variables and Wilcoxon rank-sum tests for continuous age and length of stay

**Figure:**
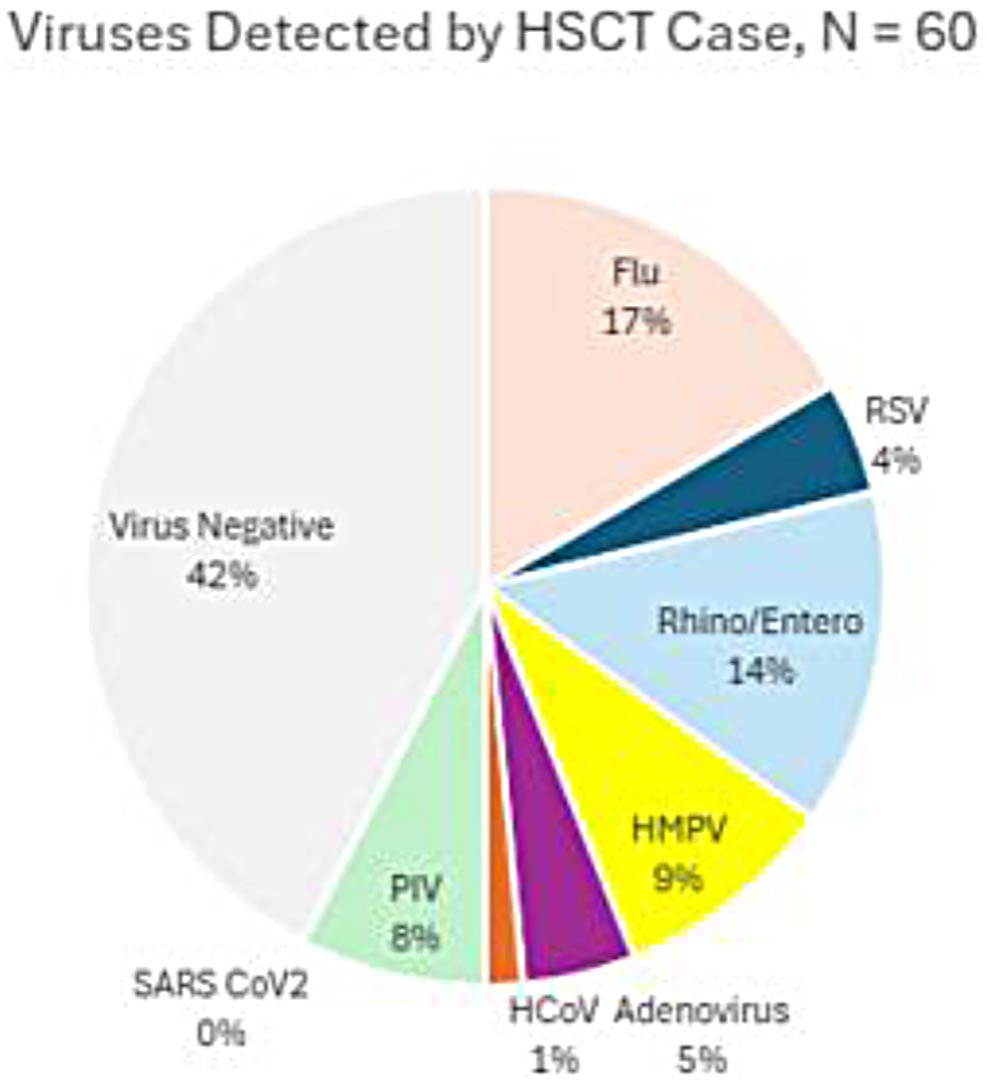
Viruses Identified Among HSCT Pediatric Cases, December 2016- September 2020

**Methods:**

This prospective, multicenter study enrolled children < 18 years with ARI across seven U.S. health systems (2016–2020). Children were enrolled if seen in the ED or hospitalized. Respiratory specimens were tested by RT-PCR for common viruses on mid-turbinate nasal swabs. Exclusion criteria included illness >14 days, non-respiratory diagnoses, recent prior hospitalization, neonates never discharged home, and neutropenic fever (ANC < 500). Children were immunocompetent if they lacked HSCT, transplant, malignancy, sickle cell disease, or other immunosuppression. Comparisons used Chi-squared and Wilcoxon rank-sum tests.

**Figure:**
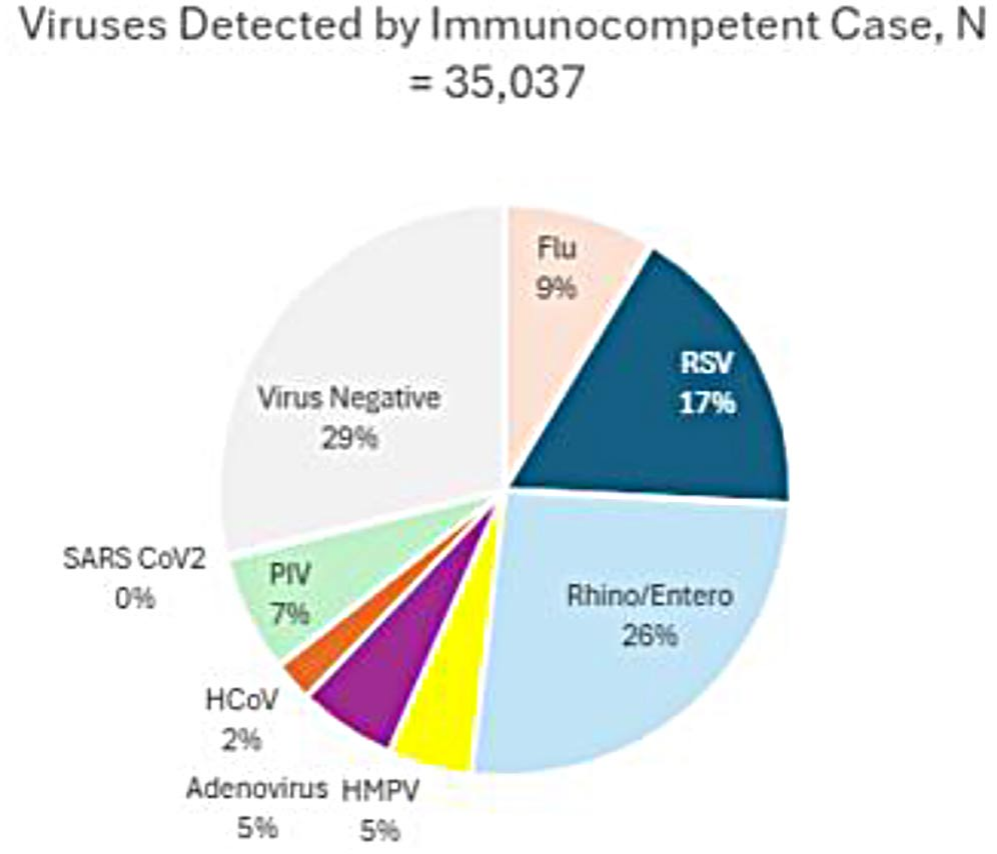
Viruses Identified Among Immunocompetent Pediatric Cases, December 2016- September 2020

**Results:**

A total of 60 HSCT and 35,037 immunocompetent children were enrolled. Median age was higher in HSCT recipients (9.5 vs. 1.9 years; p< 0.01). HSCT patients were more often hospitalized (91.7% vs. 49.0%; p < 0.01). Among 55 HSCT and 17,158 immunocompetent hospitalized patients, ICU admission (14.6% vs. 17.3%; p=0.86) and intubation (1.6% vs. 3.4%; p=0.80) rates were similar. However, mortality was higher in HSCT (1.7% vs. 0.1%; p< 0.01). Median LOS was longer in HSCT (3 vs. 2 days), with fewer discharged in 0–1 day (30.9% vs. 45.3%) and more hospitalized ≥ 5 days (25.5% vs. 13.1%; p< 0.01). Among influenza-positive patients, oseltamivir use was higher in HSCT (90.9% vs. 34.3%; p< 0.01).

**Conclusion:**

HSCT recipients with ARI were older, more frequently hospitalized, stayed longer, and had higher mortality, underscoring the need for enhanced preventive strategies in this population.

**Disclosures:**

All Authors: No reported disclosures

